# Phenotypic, Ultra-Structural, and Functional Characterization of Bovine Peripheral Blood Dendritic Cell Subsets

**DOI:** 10.1371/journal.pone.0109273

**Published:** 2014-10-08

**Authors:** Janet J. Sei, Amanda S. Ochoa, Elizabeth Bishop, John W. Barlow, William T. Golde

**Affiliations:** 1 Plum Island Animal Disease Center, Agricultural Research Service, USDA, Greenport, New York, United States of America; 2 Department of Animal Sciences, University of Vermont, Burlington, Vermont, United States of America; Beth Israel Deaconess Medical Center, Harvard Medical School, United States of America

## Abstract

Dendritic cells (DC) are multi-functional cells that bridge the gap between innate and adaptive immune systems. In bovine, significant information is lacking on the precise identity and role of peripheral blood DC subsets. In this study, we identify and characterize bovine peripheral blood DC subsets directly *ex vivo*, without further *in vitro* manipulation. Multi-color flow cytometric analysis revealed that three DC subsets could be identified. Bovine plasmacytoid DC were phenotypically identified by a unique pattern of cell surface protein expression including CD4, exhibited an extensive endoplasmic reticulum and Golgi apparatus, efficiently internalized and degraded exogenous antigen, and were the only peripheral blood cells specialized in the production of type I IFN following activation with Toll-like receptor (TLR) agonists. Conventional DC were identified by expression of a different pattern of cell surface proteins including CD11c, MHC class II, and CD80, among others, the display of extensive dendritic protrusions on their plasma membrane, expression of very high levels of MHC class II and co-stimulatory molecules, efficient internalization and degradation of exogenous antigen, and ready production of detectable levels of TNF-alpha in response to TLR activation. Our investigations also revealed a third novel DC subset that may be a precursor of conventional DC that were MHC class II^+^ and CD11c^−^. These cells exhibited a smooth plasma membrane with a rounded nucleus, produced TNF-alpha in response to TLR-activation (albeit lower than CD11c^+^ DC), and were the least efficient in internalization/degradation of exogenous antigen. These studies define three bovine blood DC subsets with distinct phenotypic and functional characteristics which can be analyzed during immune responses to pathogens and vaccinations of cattle.

## Introduction

Dendritic cells (DC) are a heterogeneous population of cells that play a critical role in initiation and linking of the innate and adaptive immune response [1]. Extensive knowledge of the phenotype and function of DC has been derived from mouse studies [2]–[6]. Analysis of human DC populations has focused on cells cultured from monocyte precursors (moDC) in the presence of cytokines [7], and mature DC, both isolated from peripheral blood [8]–[10].

In cattle, the role of DC has been investigated by assessing the function of afferent lymph veiled cells (ALVC) isolated following cannulation of lymphatic vessels [11]–[15]. Although cannulation facilitates the investigation of large numbers of DC directly *ex vivo*, it is technically demanding, taking up to six months to complete, and requires surgery which is not always logistically feasible [13]. Several studies have generated bovine moDC from monocyte precursors isolated from peripheral blood to assess their function in response to pathogen infections [16]–[20]. However, a recent study has demonstrated that investigation of *in vitro* derived moDC does not accurately represent *in vivo* populations [21]. These investigators show that *in vitro*, moDC had an increased capacity for proteolysis, a characteristic exhibited by macrophages, but not *ex vivo* isolated DC [21]. Furthermore, it has previously been demonstrated that moDC and blood DC differ in their ability to stimulate T lymphocytes [22]. Thus the physiological relevance of *in vitro* derived moDC is problematic, and caution is necessary when using moDC as a model for DC.

A few studies have investigated the phenotype and function of bovine peripheral blood DC [23]–[26]. In these studies, enrichment protocols were utilized to deplete non-DC [23]–[26]. While the DC population is enriched, a major limitation of this approach is the difficulty of entirely depleting other cell types, thus reducing the overall purity of the DC yield. Consequently, careful interpretation should be exercised when attributing DC immuno-phenotype and functions to DC enriched populations.

Peripheral blood DC have been divided into two main subsets: plasmacytoid DC (pDC) and conventional DC (cDC). pDC have been shown to produce large amounts of type I interferons (IFN) that limit virus spread, enhance antigen presentation, and increase cytotoxic function [27]–[29]. cDC function as efficient naïve T cell stimulators by presenting degraded antigenic peptides to T cells in the context of MHC molecules [1]. Additionally, cDC produce pro-inflammatory cytokines, which have potent down stream immune stimulatory function [1].

Generally, pDC in humans [28]–[30] have been shown to be CD4^+^/CD11c^−^/lineage^−^ (monocyte^−^, B cell^−^, T cell^−^, NK cell^−^). In both swine and bovine, pDC have been defined as CD4^+^/MHC class II^+^/CD172a^+^/lineage^−^ [24], [26], [31]. In contrast, cDC in humans [28], [30], [32] have been identified as CD4^−^/CD11c^+^/lineage^−^ cells. Porcine cDC [31] are defined as CD4^−^/MHC II^+^/CD80/86^+^/CD172a^+^/lineage^−^ and bovine cDC [23] as MHC II^+^, CD11c^+^/CD172a^+^/lineage^−^.

Given the limitations in the investigation of bovine DC by utilization of enrichment methods, our goal was to use multi-color flow cytometry (5–7 color) to identify bovine blood DC subsets and characterize their phenotype, morphology, and function directly *ex vivo* without any requirement for secondary *in vitro* culture. Specifically, we questioned whether DC subsets differ in their ultra-structural morphology, expression of MHC class II and co-stimulatory molecules, capabilities to mature, produce pro-inflammatory cytokines, produce type I IFN in response to toll-like receptor (TLR) agonists, and their ability to internalize and degrade exogenous antigen.

In this study, we demonstrate that three distinct DC subsets could be identified in bovine peripheral blood: one DC subset corresponds to pDC that express CD4, and two cDC populations that express MHC class II. These DC subsets have differential expression of functional cell surface markers, ultra-structural appearance, cytokine profile, and antigen processing abilities. Our characterization of bovine peripheral blood DC subsets provides insight into DC phenotype and function, which can be applied to investigations of infection, vaccination, and therapeutic intervention in cattle and thus inform the rational design of vaccines for bovine pathogens.

## Materials and Methods

### Animals

All animal experiments were performed either at University of Vermont (UVM) Burlington, VT or at Plum Island Animal Disease Center (PIADC), Plum Island, NY following protocols approved by the respective Institutional Animal Care and Use Committees (IACUC). Holstein steers used were 6 months of age and weighing 400 pounds. These cattle were either maintained at the Miller Research Complex, UVM, Burlington, VT or transferred from UVM to PIADC and acclimated for at least 1 week prior to experimentation.

### Blood sampling

Peripheral blood was drawn from the jugular vein into vacutainer tubes containing sodium heparin. For isolation of peripheral blood mononuclear cells (PBMC) via leukophoresis, blood was diluted 1:1 with PBS in 50-ml conical tubes, underlayed with Histopaque-1083 (Sigma-Aldrich, St. Louis, MO) and centrifuged at 1700×g for 30 minutes. The interphase consisting of PBMC was collected and cells were washed three times in PBS. PBMC were resuspended in RPMI-1640 (Life Technologies, Grand Island, NY), that was supplemented with 10% heat-inactivated fetal bovine serum (FBS) (Thermo Scientific, Waltham, MA), 1X L-glutamine (Life Technologies), and 1X anti-biotic/anti-mycotic (Life Technologies).

### Multi-color Flow cytometry

Total PBMC were enumerated and 1×10^6^ cells were aliquoted per well. Cells were washed once with FACS buffer (0.3% BSA, 0.9% sodium azide, PBS). All antibodies were diluted in FACS buffer and cells were incubated on ice for 20 minutes and washed twice in FACS buffer. The anti-bovine antibodies used are outlined in Table 1 and Table 2. To determine cell viability either LIVE/DEAD Fixable Yellow Dead Cell Stain Kit or LIVE/DEAD Fixable Near-IR Dead Cell Stain Kit (Life Technologies, Grand Island, NY) were used. Prior to staining cells with antibodies, cells were incubated with LIVE/DEAD stain for 30 minutes on ice and washed twice with FACS buffer. The emission of LIVE/DEAD Fixable Yellow Dead Cell Stain allowed us to detect its fluorescence using the Violet laser, Quantum-Dot 525 channel on the LSR-II (BD Biosciences, San Jose, California), whereas LIVE/DEAD Fixable Near-IR Dead Cell Stain Kit was detected using the Red laser, APC-Cy7 channel. FACS DIVA Software was used for acquisition and analysis (BD Biosciences, San Jose, California). FlowJo Software 9.6.4 Version (TreeStar, Ashland, USA) was also used for analysis.

**Table 1:** Antibody Panel for Surface Staining of Bovine Peripheral Blood DC subsets.

Antibody	Isotype	Clone	Source/Catalog #
CD205 FITC	IgG2b	CC98	AbD Serotec, Raleigh, NC
CD11c	IgM	BAQ153A	Washington State University (WSU), Pullman, WA
CD3	IgG1	MM1A	WSU, Pullman, WA
CD14	IgG1	MM61A	WSU, Pullman, WA
CD11b	IgG1	MM12A	WSU, Pullman, WA
sIgM	IgG1	IL-A30	AbD Serotec, Raleigh, NC
MHC II R-PE	IgG2a	IL-A21	AbD Serotec, Raleigh, NC
CD4 Alexa 647	IgG2a	CC8	AbD Serotec, Raleigh, NC
CD80 FITC	IgG1	IL-A159	AbD Serotec, Raleigh, NC
CD172a R-PE Cy5	IgG2b	CC149	AbD Serotec, Raleigh, NC
CD16 FITC	IgG2a	KD1	AbD Serotec, Raleigh, NC
PE-Cy7 IgM –secondary ab	Rat anti-mouse	II/41	eBiosciences, San Diego, CA
BV421 IgG1–secondary ab	Rat anti-mouse	A85-1	BD Biosciences, San Jose, California
LIVE/DEAD Fixable Near-IR Dead Cell Stain Kit			Life Technologies, Grand Island, NY
LIVE/DEAD Fixable Yellow Dead Cell Stain Kit			Life Technologies, Grand Island, NY

**Table 2:** Antibody Panel for Intracellular Cytokine Staining.

Antibody	Isotype	Clone	Source/Catalog #
TNF-a biotin	IgG2a	CC328	AbD Serotec, Raleigh, NC
CD11c	IgM	BAQ153A	WSU, Pullman, WA,
CD3	IgG1	MM1A	WSU, Pullman, WA
CD14	IgG1	MM61A	WSU, Pullman, WA
CD11b	IgG1	MM12A	WSU, Pullman, WA
sIgM	IgG1	IL-A30	AbD Serotec, Raleigh, NC
CD4 FITC	IgG2a	CC8	AbD Serotec, Raleigh, NC
PE-Cy7 IgM –secondary	Rat anti-mouse	II/41	eBiosciences, San Diego, CA
APC Cy7 IgG1–secondary	Goat ant-mouse		Southern Biotech, Birmingham, Alabama
BV421Streptavidin – secondary			Biolegend, San Diego, CA
LIVE/DEAD Fixable Near-IR Dead Cell Stain Kit			Life Technologies, Grand Island, NY

### Sorting of DC subsets

Approximately 1.5×10^9^ PBMC were isolated as described above. To deplete T cells, B cells, NK cells, and monocytes, PBMC were re-suspended in staining buffer (1X Calcium and Magnesium free PBS, 2 mM of EDTA (Life Technologies, Grand Island, NY), and 0.5% Bovine Serum Albumin Fraction V (Life Technologies, Grand Island, NY). PBMC were stained with unlabeled mouse anti-bovine CD3/IgM/CD11b/CD14 (all IgG1) antibodies for 30 minutes on ice, followed by anti-mouse IgG immuno-depletion. 10 magnetic particles per cell of BioMag goat anti-mouse IgG (Qiagen, Valencia, CA) were incubated with stained PBMC for 20 minutes on ice, agitating every 5 minutes. Magnetic separation was performed using BioMag Flask Separators (Polysciences Inc, Warrington, PA) that can hold a 75cc flask. PBMC in 75cc flasks were applied on the separator for 5 minutes, for magnetic beads to attach, and the unbound cell fraction was assessed for enrichment of DC. This enriched sample was re-suspended in staining buffer and was further stained with anti-CD11c (IgM), MHC class II–R-PE (IgG2a), and CD4–Alexa 647 (IgG2a) monoclonal antibodies for 30 minutes on ice. To detect unlabeled primary antibodies, cells were stained with BV421 anti-IgG1 and PE-Cy7 anti-IgM secondary antibodies, followed by staining with LIVE/DEAD Fixable Near-IR Dead Cell Stain Kit, both for 30 minutes on ice. Cells were sorted simultaneously using the BD FACS Aria II, three-way cell sorter, using a 100 micron nozzle (BD Biosciences, San Jose, CA).

### TLR Agonist Stimulation

Cells were stimulated with the following TLR agonists: CpG 2006 oligonucleotide human Type B – 10 µg/ml final concentration (Invivogen, San Diego, CA), CpG 2216 oligonucleotide, human Type A – 10 µg/ml final concentration (Invivogen, San Diego, CA), Polyinosine-polycytidylic acid (Poly I:C) – 25 µg/ml final concentration (Sigma-Aldrich, St. Louis, MO), Lipopolysaccharide E. coli O55: B5 Calbiochem – 10 ug/ml final concentration (EMD Millipore, Billerica, MA), R848 – 25 µg/ml final concentration (Enzo Life Sciences Inc, Farmingdale, NY). FACS sorted DC subsets were stimulated for 12 hours with TLR agonists, and assessed for expression of MHC class II and CD80. PBMC were stimulated with TLR agonists for 5 hours, and assessed for TNF-alpha production. Cells re-suspended in supplemented RPMI-1640 media were used as controls. Due to the limited number of cells isolated by FACS, only single wells were assessed for MHC class II and CD80.

### Intracellular cytokine staining

1×10^6^ stimulated and un-stimulated PBMC per well, were treated with Brefeldin A (BD Biosciences, San Jose, CA) at 1X final concentration, incubated for 5 hours at 37°C, 5% CO_2_, and assessed for TNF-alpha production via intracellular cytokine staining. PBMC were stained with LIVE/DEAD Fixable Near-IR Dead Cell Stain Kit (Life Technologies, Grand Island, NY) for 30 minutes on ice, then stained with antibodies against cell surface markers (Table 2), as described above. Cells were fixed for 10 minutes at room temperature with 1% paraformaldehyde and placed at 4°C overnight in FACS buffer. The following day, the cells were permeabilized with 1X BD FACS Perm II Buffer (BD Biosciences, San Jose, CA) at room temperature for 10 minutes, stained with biotinylated TNF-alpha (AbD Serotec, Raleigh, NC) for 20 minutes on ice. To detect the biotinylated TNF-alpha, cells were stained with BV421 labeled Streptavidin (Biolegend, San Diego, CA) for 20 minutes on ice. Acquisition and analysis was performed on a BD LSR-II using the BD DIVA software (San Jose, CA) and Flowjo Software 9.6.4 Version (TreeStar, Ashland, OR). Duplicate samples were tested.

### Transmission Electron Microscopy

FACS sorted DC subset cell pellets were fixed in Karnovsky's fixative (2.5% Glutaraldeyhyde, 1% paraformaldehyde in 0.1 M cacodylate buffer pH 7.2) for 30 minutes. After washing in 0.1 M cacodylate buffer pH 7.2, cells cells were embedded in 2% SeaPrep Agarose (Cambrex BioScience, Rockland, ME) for 15 minutes at 4°C. Crosslinking was performed again in Karnovsky's fixative for 15 minutes at 4°C. Agarose blocks were post-fixed in 1% osmium tetraoxide (OsO_4_) for 60 minutes at 4°C, then stored in 0.1 M Cacodylate Buffer overnight at 4°C. The following day, the samples were sequentially dehydrated in 35%, 50%, 70%, 85%, 95% and 100% ethanol for 10 minutes in each solution. Further dehydration was performed in propylene oxide. Blocks were infiltrated with varying ratios of propylene oxide:Spurr's Resin (3:1–30 minutes, 1:1–30 minutes, 1:3–45 minutes, 100% Spurr's –45 minutes). Samples were then embedded in fresh 100% Spurr's and allowed to polymerize overnight at 70°C. 70 nm microtome sections were cut, placed on nickel grids, and stained with 2% uranyl acetate then lead citrate. Samples were analyzed on a JEOL 1210 transmission electron microscope (JEOL, Peabody, MA) and 4000X images were obtained.

### DQ-OVA Processing

1×10^6^ PBMC were incubated at 37°C or 4°C for 1.5 hours with 2 ug/ml of DQ-OVA (Life Technologies, Grand Island, NY). Cells were washed four times with cold PBS, stained with LIVE/DEAD Fixable Yellow Dead Cell Stain, and surface marker antibodies (Table 1) as previously described. Acquisition and analysis was performed on a BD LSR-II using the BD DIVA software. Samples were tested in triplicate.

### Type I IFN Mx-CAT Assay

FACS sorted DC were stimulated with R848 as described above for 20 hours. DC re-suspended in supplemented RPMI-1640 media were used as controls. Cell suspensions were centrifuged at 700×g for 10 minutes, the supernatant was frozen at −70°C until we assessed for type I IFN production. Type I IFN was measured using an Mx promoter-chloramphenicol acetyltransferase (Mx-CAT) reporter assay as previously described [33] with a minor modification (2.5×10^5^ MDBK-t2 cells per well were used). Due to the limited number of cells isolated by FACS, only single wells were assessed for cytokine production.

### Real-time PCR analysis of TLR expression

Cells were lysed using Lysis Solution (5 PRIME, Gaithersburg, MD). Lysates were frozen at −80°C until extraction could take place. Lysates were then thawed and total RNA was purified using the PerfectPure RNA Cell & Tissue Kit (5 PRIME, Gaithersburg, MD) as per the manufacturer's instructions. Following RNA extraction, complementary DNA (cDNA) was generated using the Promega Improm-II Reverse Transcription System (Madison, WI) with a modified reaction mix. RNA template was combined with random primers. The mixture was heated to 70°C for 5 minutes using the C1000 Thermal Cycler (Bio-Rad, Hercules, CA), and then placed in ice immediately for 5 minutes. Reaction mix was then added to the RNA template. Reaction mix consisted of Improm-II 5x Reaction Mix, MgCl_2_ (25 mM), Nucleotide Mix (10 mM), rRNasin, and Improm-II Reverse Transcriptase for each cDNA synthesis reaction.

Expression of TLR3, 7, 8, 9, and GAPDH were determined by real-time PCR. GAPDH (forward, CATGTTCCAGTATGATTCCACCC; reverse GAGCTTCCCGTTCTCTGCC) [34]. TLR3 (forward, AGGCAGGTGTCCTTGAACTTG; reverse GATCTTTCAATAGATTCTGTGTTACAACGAAA) [35]. TLR7 (forward, AACTCTGCCCTGTGATGTCACTCT; reverse TGGAGAGATGCCTGCTATGTGGTT) [36]. TLR8 (forward, CCGAGCAACATGATGCAAATGGGA; reverse ATCTAAGGCACTGCCAAGACGTGA) [36]. TLR9 (forward TAATCTCCAACCGCATCCACCACT; reverse AAGGTGTTGGGCTCGATGGTCATA-5′ [34].

Reactions were carried out in optical 96-well plates using a reaction mix containing Maxima SYBR Green qPCR Master Mix (2X) (Thermo Scientific, Pittsburgh PA), forward and reverse primers (at 10 µM), and nuclease-free water. Reaction mix was combined with template master mix containing nuclease-free water and cDNA template. Amplifications were performed in a CFX96 Thermal Cycler (Bio-Rad) programmed for an initial denature of 95°C for 10 minutes, followed by 45 cycles of 95°C for 15 seconds, 60°C for 1 minute, and 72°C for 30 seconds, and imaged each cycle following 72°C extension. Relative expression of the threshold cycle (C_T_) values for each TLR was normalized to the endogenous control GAPDH, with the use of the equation 2^(−ΔC^_T_^)^, where ΔC_T_ = TLR C_T_ – GAPDH C_T_.

### Isolation of cells from secondary lymphoid organs

Retro-pharyngeal, sub-mandibular, pre-scapular, popliteal lymph nodes, and a spleen was harvested from one steer. Tissues were placed in a petri dish, and by using forceps and a scalpel, each tissue was carefully cut into small fragments weighing about 500 mg. Samples were transferred into 50 ml tubes and incubated at 37°C for 30 minutes in 0.5 mg/ml Collagenase Type I (Life Technologies, Grand Island, NY) in Hank's Balanced Salt Solution without Ca^2+^ and Mg^2+^ (Life Technologies, Grand Island, NY), while agitating every 5 minutes. Collagenase type I was inactivated by adding heat-inactivated FBS and complete RPMI-16 media. To remove clumps, the tissue was passed through a 70 µm Nylon cell strainer (BD Biosciences, San Jose, California), and using a plunger, the tissue was dissociated. Single cell suspensions were collected in a 50 ml tube and the strainer was rinsed with complete RPMI-16 to flush all the cells out of the strainer. Following centrifugation, red blood cells were lysed from the spleen sample using ACK lysing buffer (Quality Biological Inc, Gaithersburg, MD) for 10 minutes at room temperature. To remove clumps any clumps, the cell suspension were passed again through a 70 µm Nylon cell strainer. Cells were aliquot into wells, and stained with antibodies to identify DC, as described above.

## Results

### Immunophenotypic characterization of three distinct DC subsets in bovine peripheral blood

**Figure 1:**
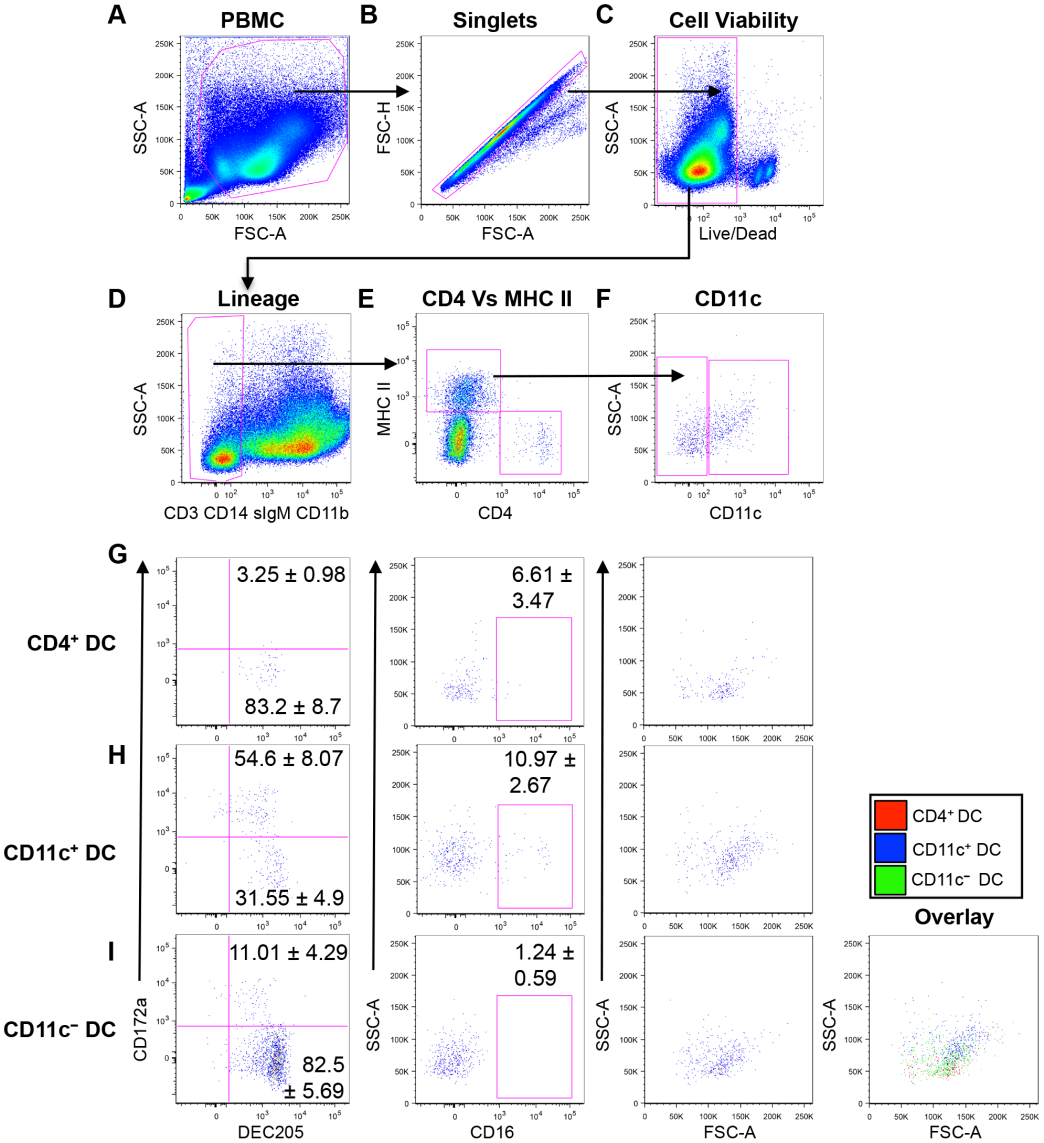
Phenotypic characterization of peripheral blood DC subsets. 7-color flow cytometric analysis of bovine PBMC to identify DC. Doublets (B) and dead cells (C) were excluded from the total PBMC population. Using lineage specific antibodies (anti-CD3, anti-CD14, anti-IgM, and anti-CD11b), T cells, monocytes, B cells and NK cells were excluded (D). Lineage negative cells were then gated to identify MHC class II^+^ and CD4^+^ cells (E), and the MHC class II^+^ cells were assessed for CD11c expression (F). Surface expression of DEC205, CD172a, and CD16 by CD4^+^ DC (G), CD11c^+^ DC (H), and CD11c^−^ DC (I). Size (FSC) and complexity (SSC) of DC subsets was also assessed by back-gating on the cell of interest. Data are representative of four independent experiments in six different animals. Numbers on plots represent average percentage of cells expressing the surface molecules in six cattle, and error bars represent standard error.

To identify and characterize peripheral blood DC subsets in cattle, we utilized seven-color flow cytometric analysis (Figure 1). The antibodies used during these experiments are outlined in Table 1. DC comprise approximately 1% of the total PBMC population making it critical to exclude both doublets (Figure 1B) and dead cells (Figure 1C) from the total PBMC population (Figure 1A), as these may produce “false positives” when identifying the small population of interest. We then used lineage specific markers to exclude T cells (CD3^+^), B cells (surface IgM^+^), monocytes (CD14^+^), and CD11b^+^ cells, which include CD14^−^ monocytes, NK cells, and B cells (Figure 1D). The resulting lineage negative population (1.3±0.2% of PBMC, n = 6) was further assessed for expression of MHC class II and CD4 (Figure 1E). We identified two populations of putative DC: CD4^−^ MHC class II^+^ (0.091±0.022% of PBMC, n = 6), and CD4^+^ MHC class II^−^ (0.0085±0.003% of PBMC, n = 6), that correspond to cDC and pDC, respectively (Figure 1E). Further analysis into the CD4^−^ MHC class II^+^ population demonstrated that two sub-populations could be distinguished based on the expression of the integrin, CD11c. The majority (70.17±3.7%) of CD4^−^ MHC class II^+^ cells were CD11c^−^ (0.065±0.015% of PBMC, n = 6), while 26.1±3.69% were CD11c^+^ (0.024±0.008% of PBMC, n = 6), (Figure 1F). For the balance of this study, we focus on three bovine peripheral blood DC subsets designated as CD4^+^ DC, CD11c^−^ DC, and CD11c^+^ DC.

To further characterize the three DC subsets, we assessed whether these cells express cell surface molecules characteristic of peripheral blood DC described in other mammalian species. These included the type I C-type lectin (DEC205), the SIRP-alpha molecule (CD172a), and the FcγRIII receptor (CD16). Cytometric analysis showed that a majority of the CD4^+^ DC were DEC205^+^/CD172a^−^ (Figure 1G, 83.2±8.64%, n = 6). Two major sub-populations of CD11c^+^ DC could be identified; a major DEC205^+^/CD172a^+^ (54.6±.07%, n = 6), and a minor DEC205^+^/CD172a^−^ (31.55±4.94%, n = 6) population (Figure 1H). CD11c^−^ DC were predominantly CD205^+^/CD172a^−^ (82.5±5.69%, n = 6), although a smaller DEC205^+^/CD172a^+^ (11.01±4.29%, n = 6) could also be detected. With regards to the expression of the FcγRIII receptor, the majority of both CD4^+^ DC (Figure 1G, middle panel) and CD11c^−^ DC did not express CD16 (Figure 1H, middle panel). In contrast, a minor population of CD11c^+^ DC was CD16^+^ (Figure 1H, 10.9±2.67%, n = 6), and about 90% of CD11c^+^ DC were CD16^−^ (Figure 1H).

To further assess whether these putative DC display lymphocytic or myeloid characteristics, we utilized the flow cytometric parameter that differentiates cells based on size (FSC) and complexity (SSC) (granularity, size of nucleus, smoothness of plasma membrane). Typically, lymphocyte-derived immune cells such as T cells and B cells are smaller and less complex than myeloid-lineage cells, which include monocytes. Therefore, lymphocyte-derived cells would be visualized as low FSC/SSC cells, while myeloid-derived cells would display higher FSC/SSC characteristics. The putative DC subsets were back-gated and analyzed based on FSC and SSC. The CD4^+^ DC population had low FSC/SSC (Figure 1G, right panel), the CD11c^+^ DC subset had high FSC/SSC characteristics (Figure 1H, right panel), and majority of the CD11c^−^ DC subset (Figure 1I, second right panel) had low FSC/SSC. The overlay plot shows that CD11c^+^ DC had the highest FSC/SSC characteristics among the DC subsets (Figure 1I, right panel), therefore demonstrating that CD11c^+^ DC are the largest and most complex of the DC subtypes.

Altogether, these data demonstrate that three distinct lineage^−^ (CD3^−^/CD14^−^/sIgM^−^/CD11b^−^) DC subsets could be identified in bovine peripheral blood. A CD4^+^/MHC class II^−^/DEC205^+^/CD172a^−^/CD16^−^ subset of lymphoid-origin consistent with the phenotype of pDC [37], a CD4^−^/MHC class II^+^/CD11c^+^/DEC205^+^/CD172a^+/−^/CD16^+/−^ subtype that is of myeloid-origin, and corresponds to cDC [28]. The third bovine blood DC subset was CD4^−^/MHC class II^+^/CD11c^−^/DEC205^+^/CD172a^−^/CD16^−^ with mainly lymphoid-lineage characteristics. Based on the expression of MHC class II and lack of CD4 by CD11c^−^ DC, we initially classified this subset as belonging to the cDC subtype. However, further analysis demonstrated that CD11c^−^ DC share phenotypic characteristics similar to the CD4^+^ DC subset, such as DEC205^+^/CD172a^−^/CD16^−^ and low FSC/SSC. Therefore, at this level, we could not definitively classify the CD11c^−^ DC as either putative cDC or pDC. Further morphological and functional analyses were required to characterize this subset.

### Purification and ultra-structural characterization of bovine DC subsets

**Figure 2:**
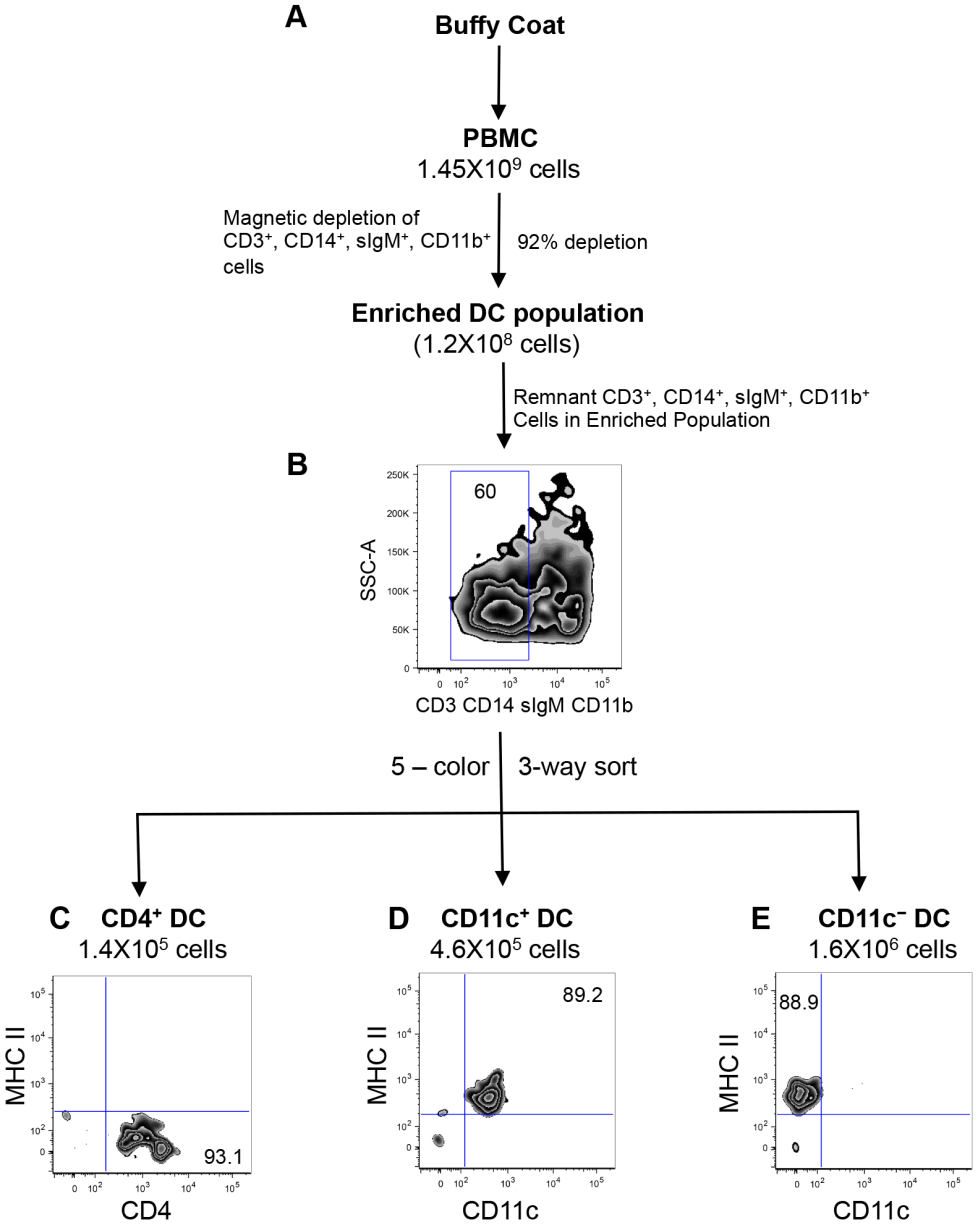
FACS purification of peripheral blood DC subsets. Schematic diagram of the DC isolation protocol from PBMC. Following density centrifugation, PBMC were subjected to immuno-magnetic depletion of lineage positive cells, to enrich DC (A). To exclude remnant lineage positive cells present in the enriched DC population, a 5-color sort was performed using a BD FACS Aria II, according to the gating strategy shown in Figure 1A – F. Three major DC subsets that are MHC class II^−^/CD4^+^ (C) MHC class II^+^/CD11c^+^ (D), and MHC class II^+^/CD11c^−^ (E). Numbers on plots represent percentage of cells. Data are representative of four independent experiments.

In order to confirm our characterization of the putative bovine blood DC subsets on the basis of morphology and function, we purified these cell populations from peripheral blood. First, PBMC were depleted of non-DCs immuno-magnetically, then the lineage negative population was sorted by fluorescence activated cell sorting (FACS) to purify DC populations from the enriched fraction (Figure 2A). Lineage positive cells were immuno-magnetically depleted by using antibodies described in Figure 1. Using this technique, we efficiently depleted 92–94% of T cells, B cells, NK cells and monocytes (Figure 2A). To determine the frequency of DC within the enriched population, we stained the cells with antibodies against CD3, sIgM, CD11b, CD14 and analyzed by flow cytometry. We found that 60% of the enriched population was lineage negative, whereas 40% were lineage positive (Figure 2B), demonstrating that even with a high immuno-magnetic depletion efficiency, we could not completely deplete T cells, B cells, NK cells and monocytes from the enriched DC population.

As previously discussed, other studies that investigated bovine blood DC subsets, utilized immuno-magnetic isolation techniques to deplete non-DC, and the resulting cells were then characterized phenotypically, ultra-structurally and functionally [23]–[25]. Given the presence of T cells, B cells, NK cells and monocytes in the enriched DC population, further purification was required to definitively attribute DC characteristics to these cells. Therefore, we proceeded with a five-color FACS strategy as outlined in Figure 1A – F, to purify the putative DC subsets. The gating strategy and post-sorting purity of blood DC subsets are shown in Figure S1. By utilizing the BD FACS Aria, we performed a three-way sort to simultaneously isolate DC subsets from PBMC, and the resulting DC populations were assessed for purity. As shown in Figure 2C, sorted CD4^+^ DC were MHC class II^−^, with a purity of 93.1%, and represented 0.01% of total PBMC. CD11c^+^ DC were MHC class II^+^, 89.2% pure, and represented 0.032% of total PBMC (Figure 2D). The CD11c^−^ DC subset was also MHC class II^+^, with a purity of 88.9%, and represented 0.11% of total PBMC. The frequencies of the sorted DC subsets closely correlated with DC frequencies obtained from unsorted PBMC (Figure 1E and F), demonstrating that we lost very few DC during the purification process.

**Figure 3:**
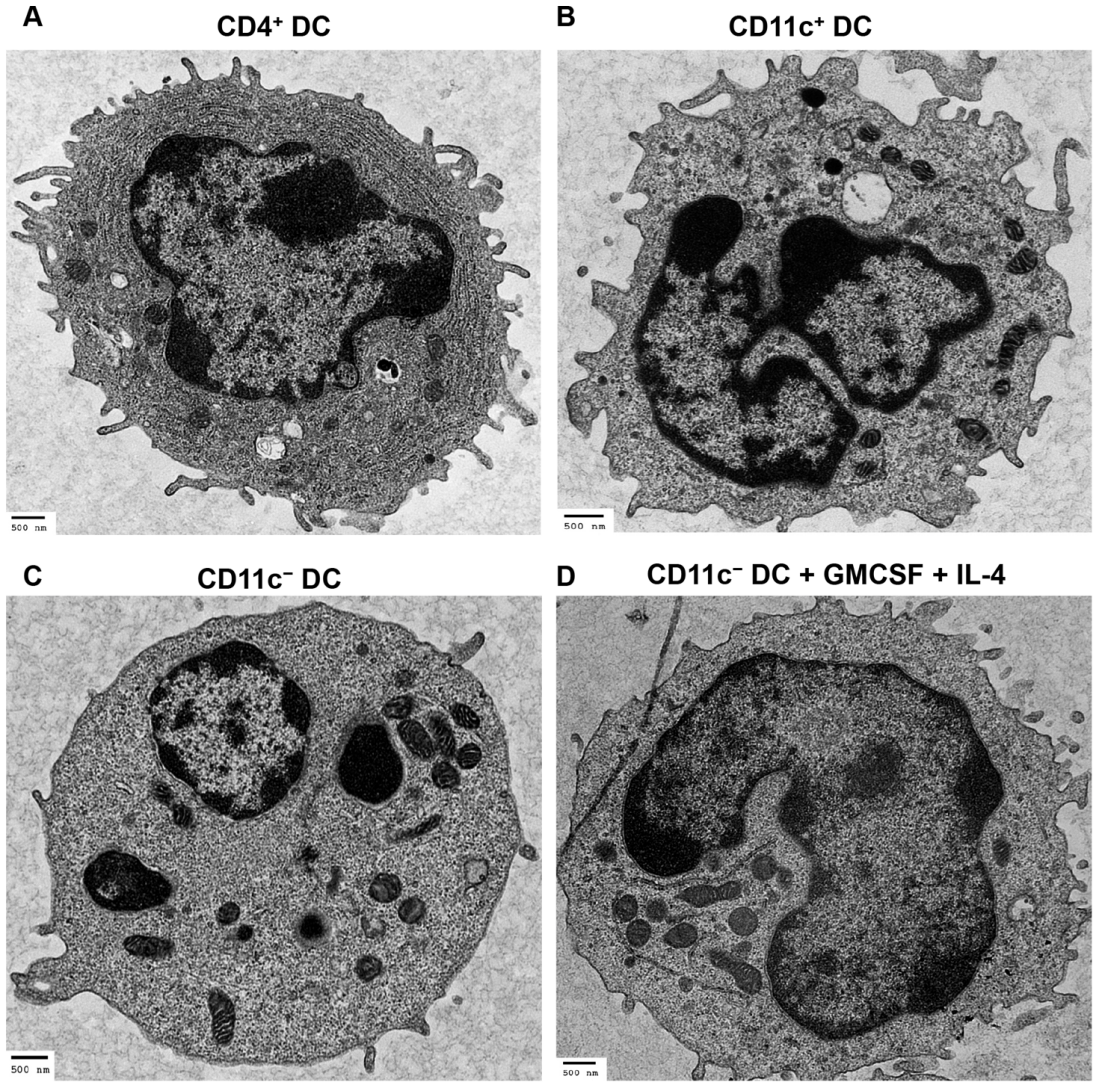
Transmission electron microscopy of peripheral blood DC subsets. TEM analyses of freshly isolated DC subsets and cytokine-stimulated CD11c^−^ DC. CD4^+^ DC display a plasmacytoid phenotype, which includes a prominent ER and dendritic protrusions on the plasma membrane (A). CD11c^+^ DC exhibit multi-lobulated nucleus, ruffled cell membrane, and do not display the prominent ER (B). CD11c^−^ DC have a smooth plasma membrane and a rounded nucleus (C). Three-day GM-CSF and IL-4-stimulated CD11c^−^ DC display dendritic projections and multi-lobulated nucleus (D). Bars denote 500 nm.

Purified DC subsets were subjected to transmission electron microscopy (TEM) for ultra-structural analysis. Morphological analysis revealed that CD4^+^ DC displayed dendritic protrusions on their cell membrane, irregular shaped nucleus, and exhibited an extensive endoplasmic reticulum (ER) and Golgi apparatus (Figure 3A). The presence of a prominent ER is a defining feature of pDC, utilized in the characteristic production of large amounts of type I IFN. In human and swine, these cells have been termed natural type I IFN producing cells [27], [28], [38].

The CD11c^+^ DC subset exhibited an irregular ruffled cell membrane populated with projections, a multi-lobulated nucleus (Figure 3B), and evidently lacked the prominent ER observed in CD4^+^ DC. The bovine CD11c^+^ DC characteristics outlined above share similarities with cDC in human blood that have a ruffled membrane, display a multi-lobulated nucleus and lack an abundant ER [28], [38]. CD11c^−^ DC displayed a smooth cell membrane, minimal plasma membrane projections, a rounded nucleus, and lacked a prominent ER (Figure 3C). Compared to CD11c^+^ DC and CD4^+^ DC, CD11c^−^ DC exhibited a previously uncharacterized, and distinct cellular morphology.

Our prior analysis shown in Figure 1, demonstrated that CD11c^−^ DC share phenotypic characteristics with both CD11c^+^ DC and CD4^+^ DC. This led us to question whether CD11c^−^ DC were precursors of either CD11c^+^ DC or CD4^+^ DC. Sorted CD11c^−^ DC were cultured for 3 days with bovine GM-CSF and bovine IL-4, which are cytokines that drive the differentiation of monocyte precursors into DC in mice, humans, cattle and swine [7], [16]–[20]. TEM analysis showed that GM-CSF and IL-4-stimulated CD11c^−^ DC displayed a ruffled cell membrane, a multi-lobulated nucleus, and lacked a pronounced ER (Figure 3D), features exhibited by CD11c^+^ DC, thereby suggesting that CD11c^−^ DC are CD11c^+^ DC precursors.

Thus far, we have demonstrated that there exists three bovine DC subtypes in peripheral blood that have distinct ultra-structural features: (i) CD4^+^ DC exhibit an extensive ER and dendritic protrusions, characteristic of pDC, (ii) CD11c^+^ DC display a ruffled cell membrane, multi-lobulated nucleus, and lack a prominent ER; indicative of cDC, and (iii) CD11c^−^ DC display a smooth cell membrane, rounded nucleus, and lack a pronounced ER. Upon stimulation with GM-CSF and IL-4, CD11c^−^ DC differentiate into cells exhibiting a ruffled cell membrane, multi-lobulated nucleus, and lack a prominent ER, all features of CD11c^+^ DC; therefore they could be considered to be cDC precursors.

### Maturation of DC subsets

**Figure 4:**
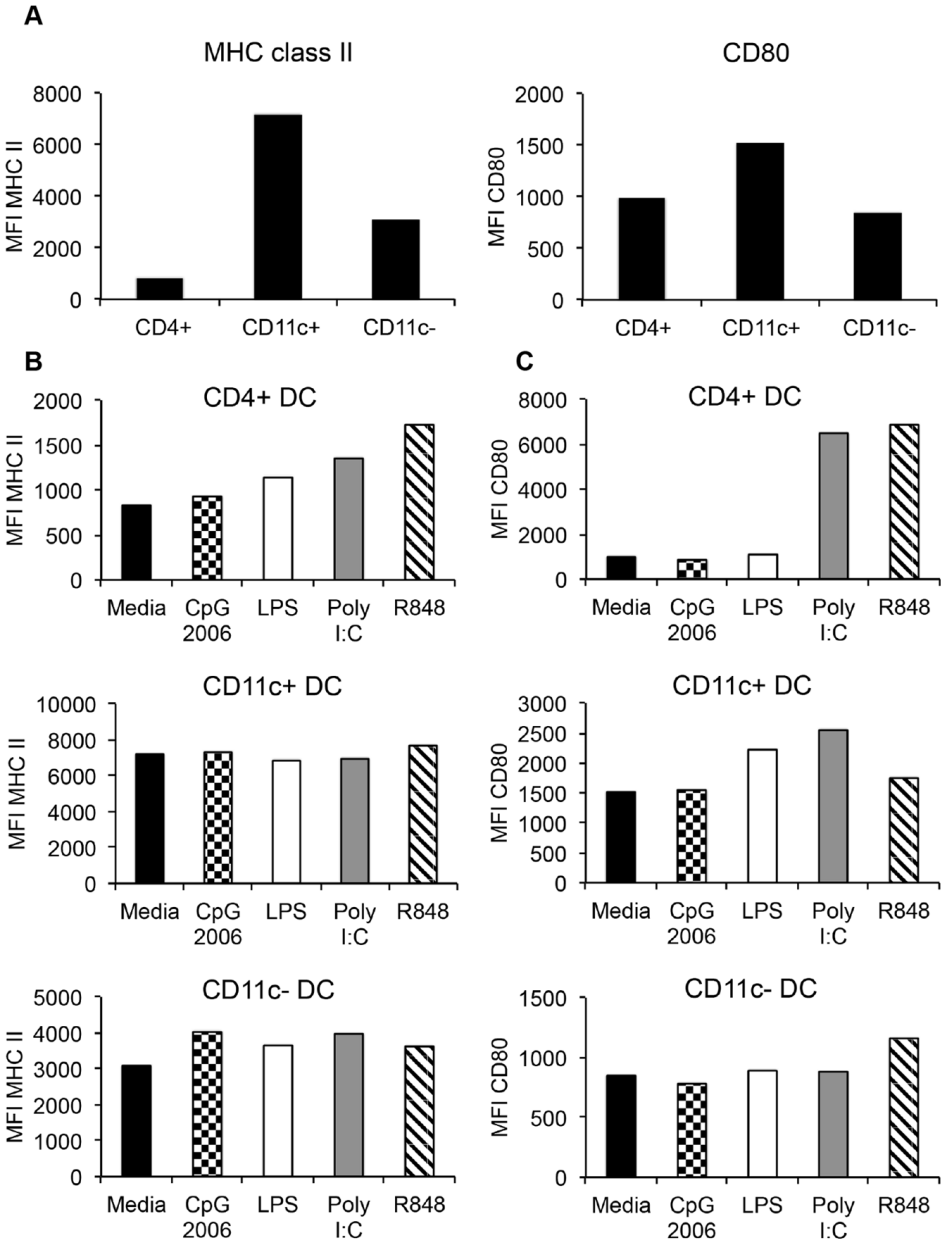
Expression levels of MHC class II and co-stimulatory molecules by un-stimulated and TLR-activated peripheral blood DC subsets. Comparison of expression of MHC class II and CD80 by un-stimulated, FACS purified CD4^+^ DC, CD11c^+^ DC, and CD11c^−^ DC (A). These DC subsets were stimulated with TLR-agonists or media control for 12 hours and subjected to immuno-staining and flow cytometric analysis. The expression of MHC class II (B) and CD80 (C) by individual DC subsets is shown following TLR-activation. Data shown is one of two experiments with virtually identical results, expressed as mean fluorescence intensity (MFI).

In the resting state, DC exist as immature cells, which are characterized by low surface expression of co-stimulatory (CD80 and CD86) and MHC class II molecules, and thus are inefficient in activating naïve T cells [39]. Once DC interact with activating agents such as pathogen associated molecular patterns (PAMP) molecules [40], they undergo “maturation” [41], [42]. DC maturation is characterized by an increase in the surface expression of co-stimulatory and MHC class II molecules [41], and the production of pro-inflammatory cytokines [42]. We therefore sought to determine the expression patterns of MHC class II and CD80 on purified immature un-stimulated DC subsets, and determine whether stimulation with various TLR agonists would induce maturation. Un-stimulated CD4^+^ DC did not express MHC class II, but expressed similar levels of CD80 as CD11c^−^ DC (Figure 4A). CD11c^+^ DC expressed the highest levels of MHC class II and CD80 (Figure 4A), whereas CD11c^−^ DC expressed high levels of MHC class II (albeit lower than CD11c^+^ DC), and expressed similar levels of CD80 as CD4^+^ DC (Figure 4A).

To induce maturation, DC were stimulated with several TLR agonists: R848 (TLR7/8), polyinosine-polycytidylic acid (Poly I:C) (TLR3), CpG-ODN (TLR9), and lipopolysaccharide (LPS) (TLR4) [43]–[45]. Each FACS sorted DC subset was stimulated with TLR agonists or media control for 12 hours, and expression levels of MHC class II and CD80 were determined by flow cytometry. CD4^+^ DC significantly up-regulated the expression of MHC class II (Figure 4B) and CD80 (Figure 4C) following stimulation with LPS, R848, and Poly I:C. CD11c^+^ DC did not up-regulate MHC class II (Figure 4B), but increased CD80 expression (Figure 4C) upon stimulation with Poly I:C, R848 and LPS. The CD11c^−^ DC had a very modest increase in the expression of MHC class II in response to all TLR agonists (Figure 4B), and an equally modest increase in CD80 expression in response to R848 (Figure 4C).

### TNF-alpha and Type I IFN production

**Figure 5:**
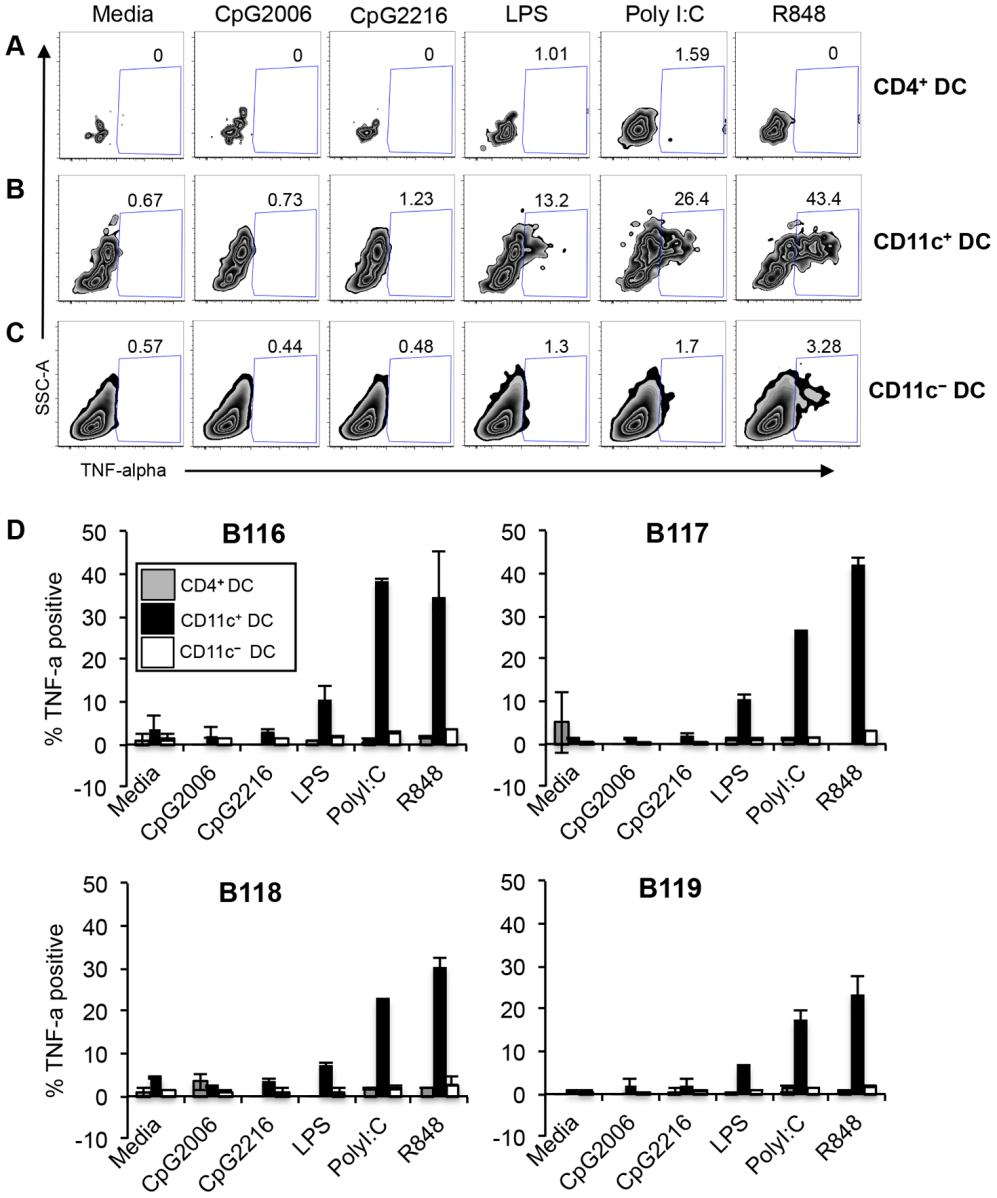
Pro-inflammatory cytokine production by peripheral blood DC subsets. PBMC were stimulated for 5 hours with TLR-agonists, and simultaneously treated with Brefeldin A. Cells were immuno-stained with antibodies against surface markers as demonstrated in Figure 1, then intracellularly stained to detect TNF-alpha production. Numbers in plots represent percentage of CD4^+^ DC (A), CD11c^+^ DC (B), and CD11c^−^ DC (C) producing TNF-alpha. Plots are representative of one animal. Graphs (D) show TNF-alpha production by DC subsets in 4 animals. Data are representative of two independent experiments. Error bars represent standard deviation.

Stimulation of DC with TLR agonists activates the transcription factor NF-κB that translocates into the nucleus to transcribe pro-inflammatory cytokines such as IL-12 and tumor-necrosis factor-alpha (TNF-alpha) [44]. We therefore examined whether DC subsets produce TNF-alpha following stimulation with the above TLR agonists. The antibody panels used during these experiments are outlined in Table 2. Figure 5A – C displays representative plots of TNF-alpha production by DC subsets from one steer. CD4^+^ DC did not produce any TNF-alpha in response to all the TLR agonists tested (Figure 5A). A significant population of CD11c^+^ DC produced TNF-alpha following stimulation with LPS (13.2%), Poly I:C (26.4%), and R848 (43.4%) compared to media control (Figure 5B). 3.28% of CD11c^−^ DC stimulation with R848 produced TNF-alpha, whereas the other TLR agonists did not stimulate TNF-alpha production (Figure 5C). Figure 5D shows comparative graphs from the 4 cattle tested, and demonstrates that DC subsets from all animals responded in a similar manner in response to TLR agonists. CD11c^+^ DC were the predominant subset that produced TNF-alpha in response to LPS, Poly I:C and R848. CD11c^−^ DC produced significantly lower levels of TNF-alpha, but only in response to R848. Lastly, CD4^+^ DC subset produced no TNF-alpha in response to any of the TLR-agonists tested (Figure 5D).

**Figure 6:**
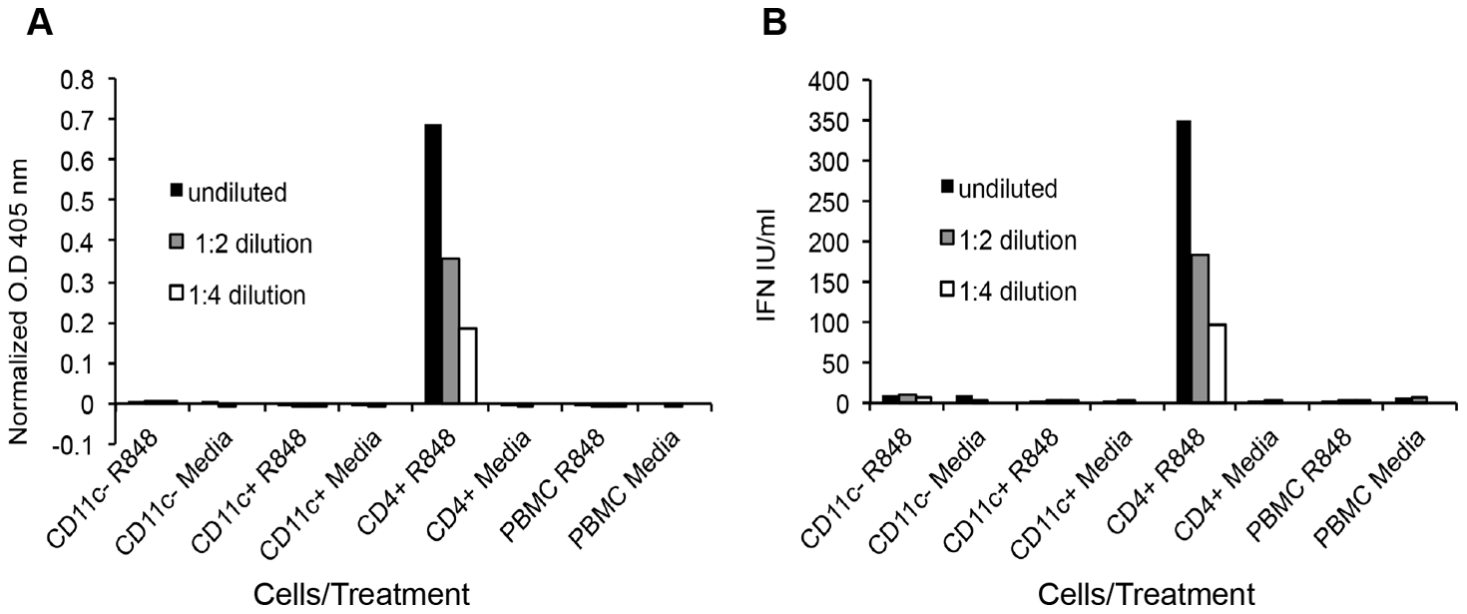
Type I IFN production by FACS purified peripheral blood DC subsets. FACS purified DC subsets and whole PBMC were stimulated with R848 or media control for 20 hours. The supernatant was assessed for the presence of type I IFN by using a Mx-CAT reporter assay. Briefly, in the presence of type I IFN, the type I IFN-inducible Mx promoter would drive the transcription of chloramphenicol acetyltransferase (CAT). CAT protein levels are then detected by an ELISA assay. CAT expression indicated by absorbance at 405 nm (A), and calculated type I IFN levels (B) are shown.

So far, our results showed that in response to TLR activation, CD4^+^ DC up-regulate MHC class II (Figure 4B) and CD80 (Figure 4C). However CD4^+^ DC did not produce TNF-alpha in response to the various TLR-agonists tested (Figure 5). Given that the primary function of pDC is to produce type I IFN [28], [38], we questioned whether the bovine peripheral CD4^+^ DC are specialized in type I IFN production. Additionally, we sought to investigate whether the other DC subsets produce type I IFN in response to TLR activation. To this end, DC subsets purified by FACS or total PBMC were stimulated for 20 hours with R848 or media control, and the supernatants were tested for type I IFN by using the Mx-chloramphenicol acetyltransferase (CAT) reporter assay, and CAT expression was detected via an ELISA assay [46]. The type I IFN producers were found to be the CD4^+^ DC population, which secreted significantly more type I IFN than the other blood cells (Figure 6A and B). Notably, type I IFN was not detected in the supernatants of TLR-activated CD11c^+^, CD11c^−^ DC, or total PBMC (Figure 6A and B).

**Figure 7:**
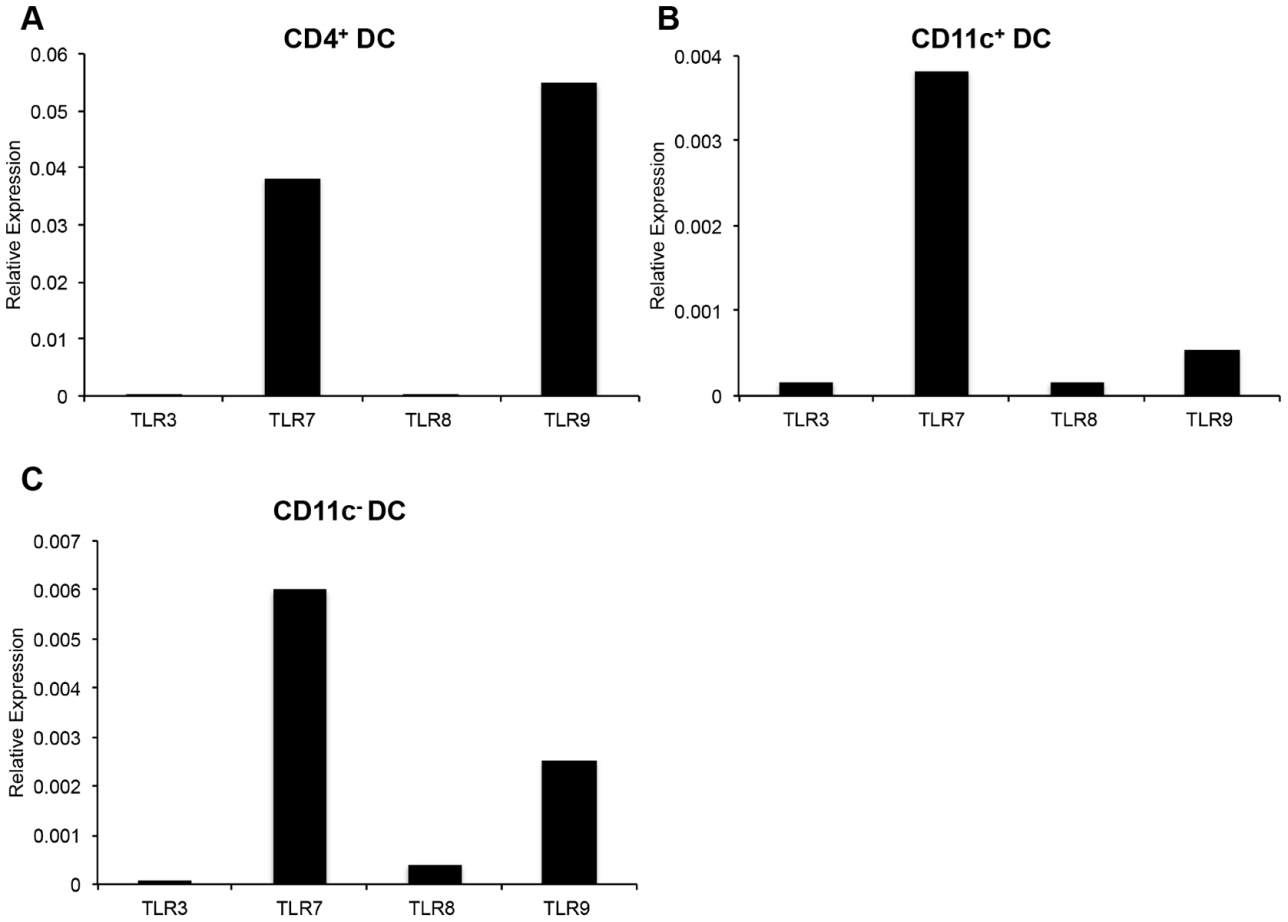
Real-time PCR quantification of TLR expression by peripheral blood DC subsets. FACS purified CD4^+^ DC (A), CD11c^+^ DC (B), and CD11c^−^ DC (C) were assessed for the expression of TLR3, TLR7, TLR8, and TLR9. Quantification of TLR expression was normalized to expression of GAPDH expression by DC subsets. Data are representative of two independent experiments.

As demonstrated above, DC maturation, production of type I IFN, and TNF-alpha can be stimulated by PAMP molecules via TLRs. Given that we observed a differential response by the TLR agonists tested, we quantified the expression levels of TLR3, TLR7, TLR8, TLR9 in FACS-sorted un-stimulated DC subsets. CD4^+^ DC expressed high levels of TLR7 and TLR9, whereas little to no expression of TLR3 or TLR8 was detected (Figure 7A). CD11c^+^ DC expressed very high levels of TLR7, and low levels of TLR3, TLR8, and TLR9 (Figure 7B). CD11c^−^ DC expressed high levels of TLR7 and TLR9, and lower levels of TLR8 (Figure 7C).

Altogether these data demonstrate that: (i) Immature CD4^+^ DC do not express MHC class II, but express CD80 (albeit lower than CD11c^+^ DC). Upon TLR-stimulation, CD4^+^ DC up-regulate both MHC class II and CD80, do not produce TNF-alpha, but produce large amounts of type I IFN. CD4^+^ DC mainly express TLR7 and TLR9. (ii) Immature CD11c^+^ DC express higher levels of MHC class II and CD80 compared to the other DC subsets. Upon stimulation with TLR-agonists, CD11c^+^ DC up-regulate the expression of MHC class II and CD80, produce the highest levels of TNF-alpha, but do not produce type I IFN. With regards to TLR expression, CD11c^+^ DC express TLR3, TLR7, TLR8, and TLR9. (iii) Immature CD11c^−^ DC express high levels of MHC class II (albeit lower than CD11c^+^ DC), and lower levels of CD80 as compared to CD11c^+^ DC. When stimulated with TLR agonists, CD11c^−^ DC modestly up-regulate the expression of MHC class II and CD80, produce TNF-alpha but significantly lower than CD11c^+^ DC, and do not produce type I IFN in response to TLR stimulation. Lastly, CD11c^−^ DC mainly expressed TLR7, TLR8, and TLR9.

### Antigen processing by bovine peripheral blood DC subsets

A primary function of DC is to internalize exogenous antigen, degrade the antigen and present peptides on MHC molecules to naïve T cells. To test the ability of bovine peripheral blood DC subsets in processing antigen, we stimulated PBMC with DQ-OVA, which is a self-quench conjugate of ovalbumin (OVA) [47], [48]. Upon successful receptor-mediated internalization and proteolytic degradation of DQ-OVA, the BODIPY FL dye that had previously been quenched, becomes fluorescent and can be detected via flow cytometry. PBMC from three cattle were stimulated with DQ-OVA for 1.5 hours and incubated at 37°C. Negative control samples were either incubated in media at 37°C or at 4°C to attest that proteolytic cleavage of DQ-OVA is a biologically active process.

**Figure 8:**
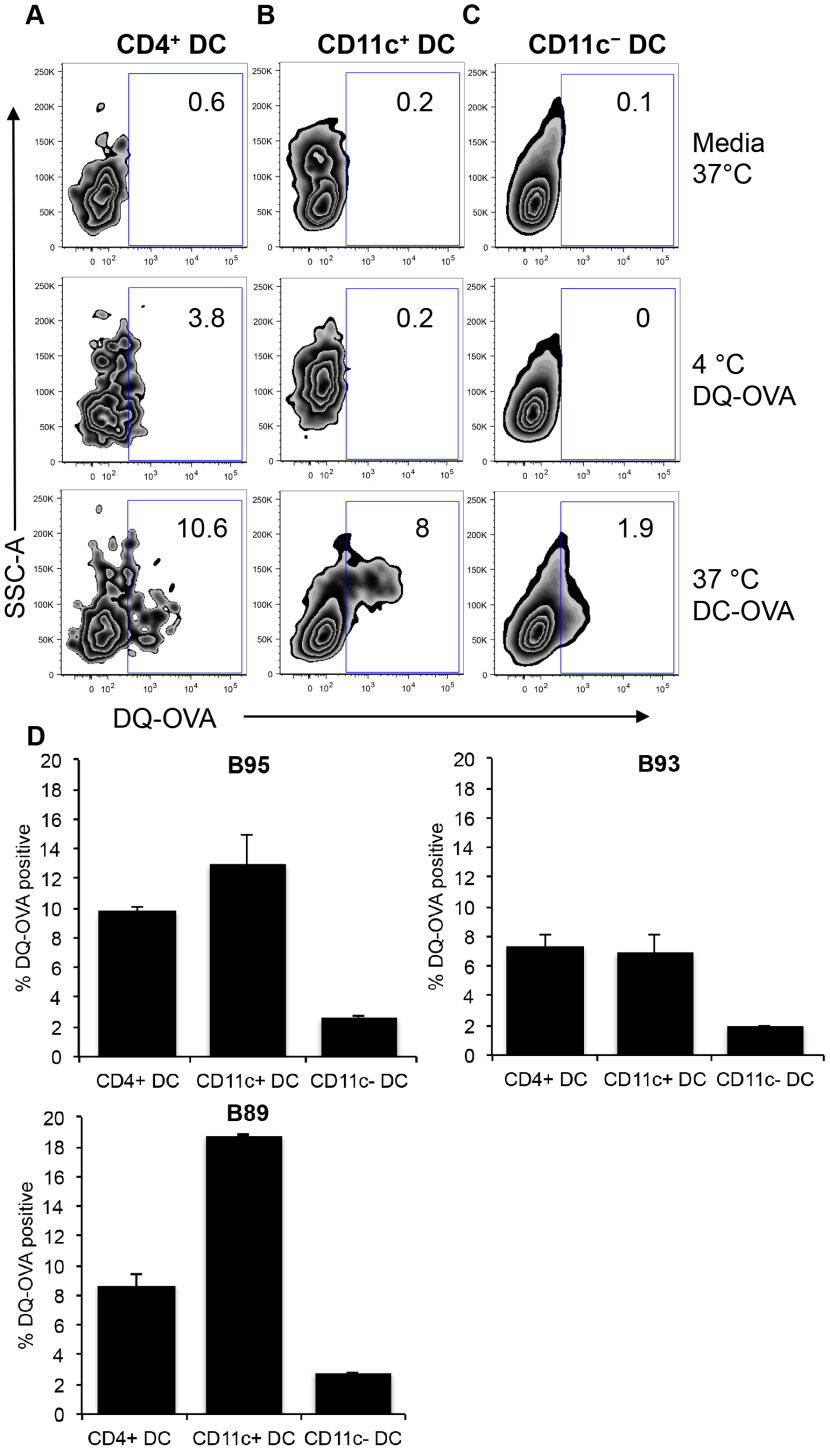
Internalization and degradation of exogenous antigen by peripheral blood DC subsets. PBMC were incubated with self-quench fluorescent DQ-OVA for 1.5 hours at 4°C and 37°C. Cells were immuno-stained with surface antibodies to identify DC subsets as outlined in Figure 1. Dot plots show fluorescence of BODIPY that represents cleavage of DQ-OVA by CD4^+^ DC (A), CD11c^+^ DC (B), and CD11c^−^ DC (C) from one animal. Calculation of DQ-OVA degradation efficiency by 3 different cattle was performed by subtracting BODIPY fluorescence of 4°C from 37°C (D). Error bars represent standard deviation.

Representative dot plots of BODIPY FL fluorescence as a measure of DQ-OVA cleavage by DC subsets from one steer are displayed in Figure 8 (A – C). For all DC subsets, no BODIPY FL fluorescence could be detected in media and 4°C controls (Figure 8A – C), although a higher 3.8% background level of BODIPY FL could be detected in CD4^+^ DC incubated at 4°C (Figure 8A). At 37°C, DQ-OVA was successfully cleaved leading to detection of BODIPY FL in 10.6% of CD4^+^ DC (Figure 8A), 8% of CD11c^+^ DC (Figure 8B), and 1.9% of CD11c^−^ DC (Figure 8C). For graphical comparison of DQ-OVA cleavage by DC subsets from three different cattle, BODIPY FL fluorescence obtained from background 4°C incubations were subtracted from cells stimulated with DQ-OVA at 37°C. We report that there was no significant difference between CD4^+^ DC and CD11c^+^ DC in DQ-OVA proteolysis, whereas the CD11c^−^ DC was least efficient in proteolytic cleavage of DQ-OVA (Figure 8D). However, in one animal, the cleavage of DQ-OVA was highest by CD11c^+^ DC (Figure 8D, right panel).

### DC subsets in secondary lymphoid organs

**Figure 9:**
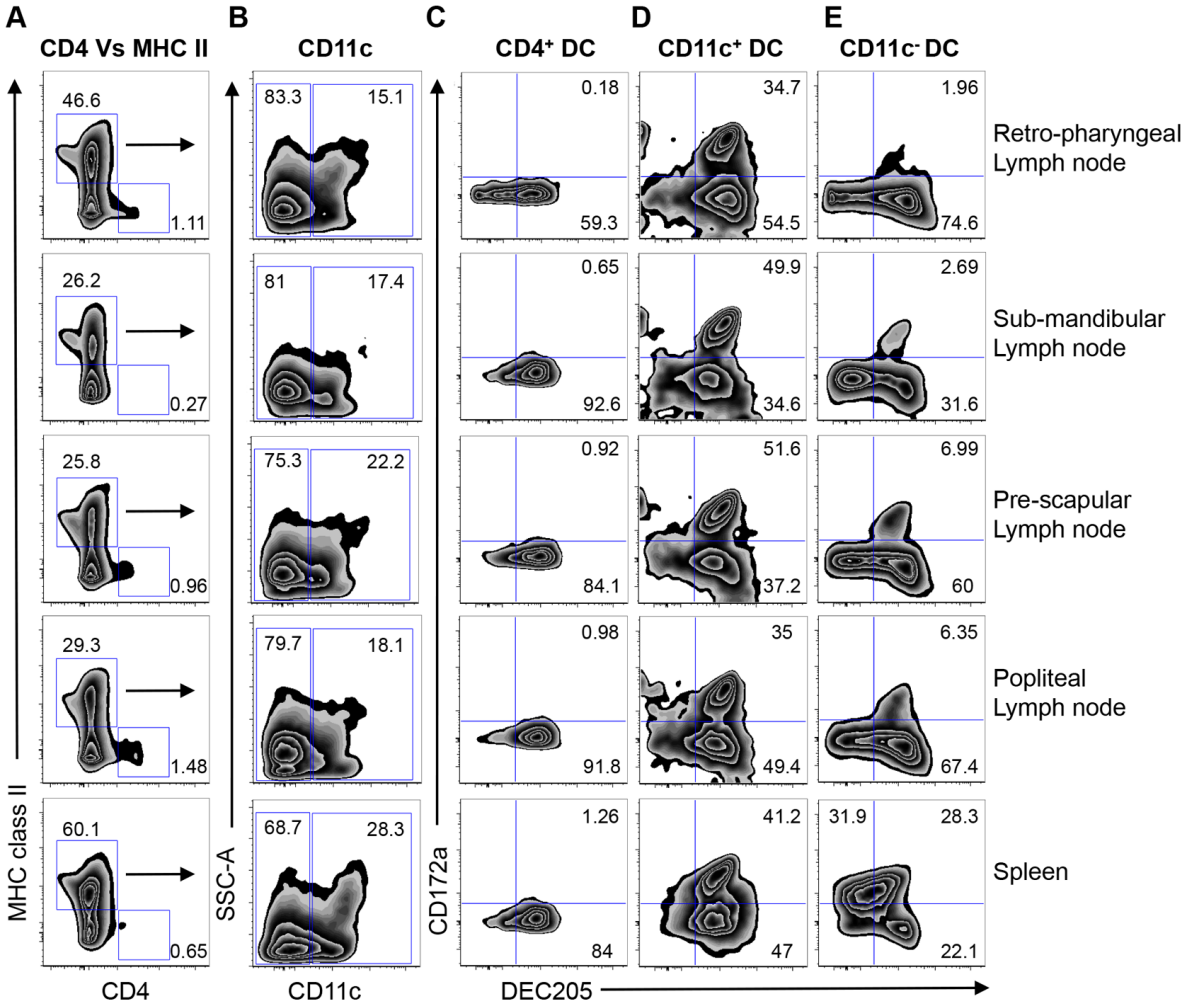
Phenotypic characterization of DC subsets in secondary lymphoid organs. Single cell suspensions from retro-pharyngeal, sub-mandibular, pre-scapular, popliteal lymph nodes, and a spleen were prepared, and 7-color flow cytometric analysis performed to identify DC. Doublets, dead cells, and lineage positive cells (T cells, monocytes, B cells and NK cells) were excluded as described in Figure 1. Lineage negative cells were then gated to identify MHC class II^+^ and CD4^+^ cells (A), and the MHC class II^+^ CD4^−^ cells were assessed for CD11c expression (B). Surface expression of DEC205 and CD172a by CD4^+^ MHC class II^−^ DC (C), MHC class II^+^/CD4^−^/CD11c^+^ DC (D), and MHC class II^+^/CD4^−^/CD11c^−^ DC (E) were assessed. Numbers on plots represent percentage of cells expressing the surface markers shown.

We next sought to investigate whether the phenotypic characterization of the blood DC subsets shown in our studies was similar to DC subsets in lymph nodes and spleens of cattle. The following peripheral lymph nodes were harvested from a naïve steer: retro-pharyngeal, sub-mandibular, pre-scapular, and popliteal. Single cell suspensions were obtained and stained for DC as demonstrated in Figure 1, and 7-color flow analysis performed. As described in Figure 1, we excluded doublets, dead cells, and lineage positive populations, then identified DC based on CD4 and MHC class II expression (Figure 9A). Similar to blood CD4^+^ MHC class II^−^ DC that constituted 0.873±0.32% of lineage negative cells, CD4^+^ MHC class II^−^ DC in the secondary lymphoid organs were 0.894±0.46% of lineage^−^ cells (Figure 9A). We observed a large population of MHC class II^+^ CD4^−^ cells (37.6±15.2% of lineage negative cells), from which CD11c expression was determined (Figure 9B). As with blood CD11c^−^ DC and CD11c^+^ DC, a larger CD11c^−^ DC (77.6±5.77%) was observed in secondary lymphoid organs, compared to 20.22±5.19% of CD11c^+^ DC. We then assessed the DC subsets for CD172a and DEC205 expression (Figure 9C – E). Our results demonstrate that similar to blood DC subsets, a majority of splenic and lymph node CD4^+^ DC were DEC205^+^ CD172a^−^ (Figure 9C, 82.36±13.52%). Comparable to blood CD11c^+^ DC, two sub-populations of CD11c^+^ DC could be identified in the secondary lymphoid organs: DEC205^+^ CD172a^−^ (44.54±8.39%) and CD172a^+^/DEC205^+^ (42.48±8%) (Figure 9D). Unlike peripheral blood CD11c^−^ DC that had a largely DEC205^+^/CD172a^−^ homogenous population (Figure 1I, 82.5±5.69%, n = 6), CD11c^−^ DC in the secondary lymphoid organs were heterogeneous. In the retro-pharyngeal, pre-scapular, and popliteal lymph nodes, there was a larger DEC205^+^ CD172a^−^ population (Figure 9E, 67.3±7.3%), and a smaller DEC205^+^ CD172a^+^ subset (4.5±2.54%) (Figure 9E). Notably, three major splenic CD11c^−^ DC sub-populations could be identified: CD172a^+^/DEC205^−^ (31.9%), CD172a^+^/DEC205^+^ (28.3%), and DEC205^+^/CD172a^−^ (22.1%).

## Discussion

Dendritic cells comprise a heterogeneous population of cells, which differ in their phenotype and function in response to pathogens. In this study, we characterized three DC subsets present in bovine peripheral blood. This work significantly extends previous studies that had investigated bovine cells exhibiting DC-like characteristics within the lineage negative peripheral blood cell population [23]–[26], [49]. These three blood DC subsets exhibit differences in their phenotype, antigen processing ability, ultra-structural morphology, TLR-induced maturation, and cytokine production. These data suggest that upon extravasation of blood DC subsets into lymphoid and non-lymphoid sites, functionally divergent DC subtypes may arise.

The first bovine peripheral blood DC subset that we identified corresponds to plasmacytoid DC [27], [28], [31], [37], [38], [50], [51]. Phenotypically, this bovine subset is defined as CD4^+^/CD3^−^/CD14^−^/CD11b^−^/IgM^−^/MHC class II^−^/CD80^med^/DEC205^+^/CD172a^−^/CD11c^−^/CD16^−^. The major characteristics that distinguished this subset as pDC was the presence of an extensive ER, and their ability to produce large amounts of type I IFN in response to TLR-stimulation. While our studies provide further insight into the phenotype of the type I IFN producing cells within the bovine lineage negative PBMC population, there appears to be differences in the nature of TLR-agonists that stimulate these cells. We report that bovine blood CD4^+^ pDC mainly expressed TLR7 and TLR9, demonstrating that bovine pDC can be stimulated by both ssRNA and dsDNA molecules, respectively. Of note is our result showing the TLR7/8 agonist R848 stimulated type I IFN production, whereas, two other studies reported that CpG motifs stimulate lineage negative cells to produce type I IFN [24], [49]. Additionally, it has previously been reported that type I IFN producing bovine lineage negative cells express TLR3 and TLR7, but not TLR9 [24]. As previously discussed, lineage negative cells consist of a heterogeneous population, thus TLR gene expression studies may have included other blood cells within the enriched population. Notably, our results analyzing sorted cells are similar to human studies which showed that blood pDC only express TLR7 and TLR9 [37].

The expression of CD172a by CD4^+^ pDC has previously been utilized as a means to identify the type I IFN producing cell [24], [26], [31], [52], [55], [56]. CD172a is a molecule that is mainly expressed by myeloid cells such as monocytes and DC [57]–[59]. Therefore, expression of CD172a by pDC was interpreted as an indication that pDC shared both lymphocytic and myeloid characteristics. In contrast to the porcine studies, we found that bovine blood pDC lack the expression of CD172a (Figure 1 and Figure 9), suggesting that bovine blood pDC do not belong to the myeloid lineage of cells. Indeed, the light scatter (FSC and SSC) of CD4^+^ pDC demonstrated that they display similar complexity and size as lymphocytes, suggesting that they belong to the lymphocyte lineage of cells. Previous investigations of pDC in humans found that pDC shared numerous features with lymphocytes, thus the term plasmacytoid T cells or T cell associated plasma cells [27], [37]. Again, one possible explanation for this discrepancy is the lack of efficiency in magnetically depleting non-DC/lineage^+^ populations and the potential contamination of the DC/lineage negative population being analyzed with lineage + cells [24], [26], [31], [52], [55]. As demonstrated in our study, immuno-magnetic depletion of non-DC does not completely eliminate lineage positive populations from PBMC (Figure 2). Our approach in characterizing pDC utilized 7 – color flow cytometric analysis to directly examine the phenotypic markers simultaneously expressed by peripheral blood pDC *ex vivo*. By gating out lineage negative cells, we eliminated the chances of phenotyping non-pDC cell populations.

Another issue illuminated by our finding is that peripheral blood pDC did not produce TNF-alpha following stimulation with any TLR-agonists, including CpG-ODN 2216 and 2006. A previous study detected TNF-alpha production by CpG-ODN 2216-stimulated porcine blood pDC, and little to no TNF-alpha in response to CpG-ODN 2006 [55]. A likely explanation of this disparity is that pDC functions in cattle and swine may have diverged. Additionally, Guzylack-Piriou et .al. incubated enriched pDC with ODN and quantified TNF-alpha protein levels via ELISA [55]. After 24-hours, both type I IFN and TNF-alpha cytokines were detected in the supernatants [55]. Given that type I IFN have been shown to stimulate the production of pro-inflammatory cytokines [60], a further possibility to consider is that type I IFN may have induced porcine pDC to produce TNF-alpha.

DEC205 is a C-type lectin that is expressed at different levels by DC, macrophages, B cells and T cells [13], [14], [61]–[63], and is involved in the internalization of CpG motifs [64], apoptotic and necrotic cells [65], [66]. Similar to bovine ALVC [13], [14], we report that bovine blood CD4^+^ pDC expressed DEC205. Indeed, bovine blood pDC were highly efficient in the internalization and proteolytic cleavage of exogenous antigen, which then stimulates intracellular signaling cascades that result in type I IFN production and pDC maturation.

Type I IFN has been demonstrated to promote exogenous antigen presentation to naïve CD4^+^ and CD8^+^ T cells by stimulating DC maturation, the production of pro-inflammatory cytokines, an increase in antigen retention, the induction of apoptosis of virus-infected cells, and enhancing internalization of apoptotic cells by DC [60], [67]–[69]. Thus, type I IFN production by bovine blood pDC following TLR ligation may induce the up-regulation of MHC class II and co-stimulatory molecules, as we describe herein, which allows pDC to transition from a poor antigen presenting cell to a potent stimulator of naïve T cells. Indeed, in mice, TLR-matured pDC have been shown to be capable of stimulating naïve T cells [70]. The production of type I IFN by pDC in response to ssRNA viruses such as foot-and-mouth virus (FMDV) [26], [52], and classical swine fever virus [53], has been demonstrated to require the internalization of immune complexes. CD32-expressing cells mediate the internalization of these immune complexes [26], [52], [53]. In addition to CD32, CD16 a low affinity IgG receptor that is expressed by NK cells, monocytes, neutrophils and eosinophils, also facilitates internalization of immune complexes [54]. While we did not assess the expression of CD32, our finding that a very small fraction of bovine blood pDC express CD16 is consistent with the idea that CD32 is the major Fc receptor involved in internalization of immune complexes [26], [52], [53].

The second bovine peripheral DC subset we characterized corresponds to conventional dendritic cells (cDC) [23], [28], [31], [38], [50]. This DC subset is identified as CD11c^+^/CD4^−^/CD3^−^/CD14^−^/CD11b^−^/IgM^−^/MHC class II^+^/CD80^+^/DEC205^+^/CD172a^+/−^/CD16^−/+^. A previous report had phenotypically characterized a bovine peripheral blood DC within the lineage^−^ population that expresses CD11c^+^/CD172a^+^, however, functional studies were not performed [23]. Consequently, data presented in this study, extends our understanding of the role of bovine blood cDC *in vivo*. We report that in their immature state, bovine blood CD11c^+^ cDC expressed the highest levels of MHC class II and co-stimulatory molecules relative to the other DC subsets. Upon TLR-stimulation, blood cDC up-regulated CD80 expression and produce large amounts of TNF-alpha. Additionally, CD11c^+^ cDC are highly efficient in the internalization and degradation of exogenous antigen. Similar to pDC, the internalization of antigen may have been facilitated by DEC205, which we found is highly expressed by this DC subset. This is likely aided by the multiple projections found on the plasma membrane, increasing the surface area required for internalization of antigen. In mice, conventional DC in lymphoid organs share similar phenotypic and functional characteristics as bovine blood CD11c^+^ DC, such as expression of DEC205, high expression levels of class II MHC and co-stimulatory molecule, and production of pro-inflammatory cytokines [4], [63], [71]. We also report that a small fraction of bovine blood, cDC express CD16 which may enhance their ability in the internalization of immune complexes.

Notably, of the three bovine blood DC subsets, CD11c^+^ cDC expressed the highest levels of the myeloid-specific marker, CD172a [57], [58], indicating that this subset belongs to the myeloid lineage of cells. Further evidence suggesting their myeloid lineage is the finding that, similar to monocytes, cDC have high FSC/SSC profiles. Indeed, ultra-structural morphological analysis showed that CD11c^+^ cDC exhibited a ruffled plasma membrane and multi-lobulated nucleus. CD172a has also been demonstrated to facilitate the binding of cells to CD4^+^ T cells [58]. Therefore, CD172a may enhance the interaction between bovine CD11c^+^ cDC and naïve CD4^+^ T cells.

The final bovine blood DC subtype that we characterized was a novel subset that can be identified as CD11c^−^/CD4^−^/CD3^−^/CD14^−^/CD11b^−^/IgM^−^/MHC class II^+^/CD80^med^/DEC205^+^/CD172a^−^/CD16^−^. Because CD11c^−^ DC shared phenotypic characteristics with both CD4^+^ pDC (lineage^−^/CD80^med^/DEC205^+^/CD172a^−^/CD16^−^) and CD11c^+^ cDC (lineage^−^/CD4^−^/DEC205^+^/MHC class II^+^), it was difficult to categorize this DC subset as belonging to either pDC or cDC. Consequently, we conducted further functional and ultra-structural analyses. Surprisingly, CD11c^−^ DC did not share morphological characteristics with either CD4^+^ pDC or CD11c^+^ cDC, suggesting that they were either a distinct sub-population of blood DC or a DC precursor that required either growth factors or cytokines to fully differentiate. Interestingly, treatment of CD11c^−^ DC with GM-CSF and IL-4 induced their differentiation into cells that morphologically resembled CD11c^+^ cDC, indicating that CD11c^−^ DC were related to the CD11c^+^ cDC lineage. TLR activation of CD11c^−^ DC led to the production of TNF-alpha, but not type I IFN, also consistent with the concept that CD11c^−^ DC are functionally similar to CD11c^+^ cDC. However, the percentage of CD11c^−^ DC producing TNF-alpha was significantly lower than CD11c^+^ cDC and are the least efficient in the internalization and degradation of exogenous antigen, suggesting that CD11c^−^ DC may require pre-stimulation in order to function as efficiently as CD11c^+^ cDC. Their inability to internalize antigen may be due to our finding that these cells exhibit a smooth plasma membrane, and lack cell membrane projections that were present in the other blood pDC and cDC. Whether stimulation with GM-CSF and IL-4 would enhance the efficiency of antigen uptake and degradation, and TNF-alpha production, needs further investigation. Given that GM-CSF and IL-4 stimulated the formation of membrane ruffles by CD11c^−^ DC, it is highly plausible that pre-activation of these cells would enhance their function.

In both human and mice, several sub-populations of DC have been described in numerous tissues including skin, gut, spleens, lungs and lymph nodes [72], [73]. In bovine, previous studies have isolated two cDC subsets from lymph fluid: a major CD172a^+^ CD11a^−^ CD13^−^ subset, and a minor CD172a^−^ CD11a^+^ CD13^+^ subtype [11], [12], [15], [74]. Functional studies showed that the former cDC subtype are potent stimulators of both CD4^+^ and CD8^+^ T cell-mediated proliferative responses [12], [74] and predominantly produce IL-1 alpha and IL-10 upon stimulation [15]. The latter DC subset mainly activates CD4^+^ T cells [12], [74] and produces IL-12 in response to stimulation [15]. Our studies demonstrate that, within secondary lymphoid organs, a notable complexity exists in the heterogeneity of cDC subsets, which differ in CD11c, DEC205, CD172a expression. Other than the predominant DEC205^+^ CD172a^−^ CD11c^−^ DC sub-population found in peripheral lymph nodes, we also observed a DEC205^+^ CD172a^+^ subtype in the secondary lymphoid organs and a CD172a^+^ DEC205^−^ population only found in the spleen tissue. Whether there exists any functional differences amongst these CD11c^−^ DC sub-populations and whether these cells stimulate different T cell responses, warrants further investigation.

Furthermore, CD11c^+^ cDC in the secondary lymphoid organs could be divided into two sub-populations: DEC205^+^ CD172a^−^ and DEC205^+^ CD172a^+^. Given that our studies also demonstrated that phenotypically similar DC subsets to blood DC could be identified in peripheral lymph nodes and spleens, the question as to whether blood DC subsets migrate into these tissues, and perform similar functions, needs additional investigation. In humans, a recent study reported that splenic and blood DC subsets share similar phenotype and function [32]. Further analyses are also required to verify whether DC subsets identified in our study, are similar to afferent lymph DC sub-populations investigated in previous studies [12], [15], [74].

In conclusion, here we describe three bovine peripheral blood DC subsets that differ phenotypically, morphologically, and functionally. We identified CD4^+^ pDC that are specialized in type I IFN production, CD11c^+^ cDC that may be specialized in naïve T cell activation as evidenced by high expression of MHC class II and CD80, production of TNF-alpha, and enhanced antigen processing capacity, and a third novel CD11c^−^ DC population that is a precursor of CD11c^+^ cDC. Our characterization of these cells will now allow for their study during infection and vaccination.

## Supporting Information

Figure S1 **Sorting Strategy for DC subsets.** PBMC were sorted on a FACS-Aria (BD, San Jose, CA) by gating on cells with appropriate forward and side scatter (rows 1 and 2), excluding dead cells (row 3), excluding lineage cells (row 4), and separating cells by expression of MHC class II, CD4, and CD11c (rows 5 and 6). Three populations were isolated as labeled; CD4+ DC, CD11c+ DC and CD11c- DC. The percent of the population isolated with the indicated phenotype are labeled in the dot plots.
(TIF)
