# Supplementary figures and images for: Phenotypic, Ultra-Structural, and Functional Characterization of Bovine Peripheral Blood Dendritic Cell Subsets
Source: PLoS One. 2014 Oct 8;9(10):e109273. doi: 10.1371/journal.pone.0109273 (PMC4190170; doi:10.1371/journal.pone.0109273)

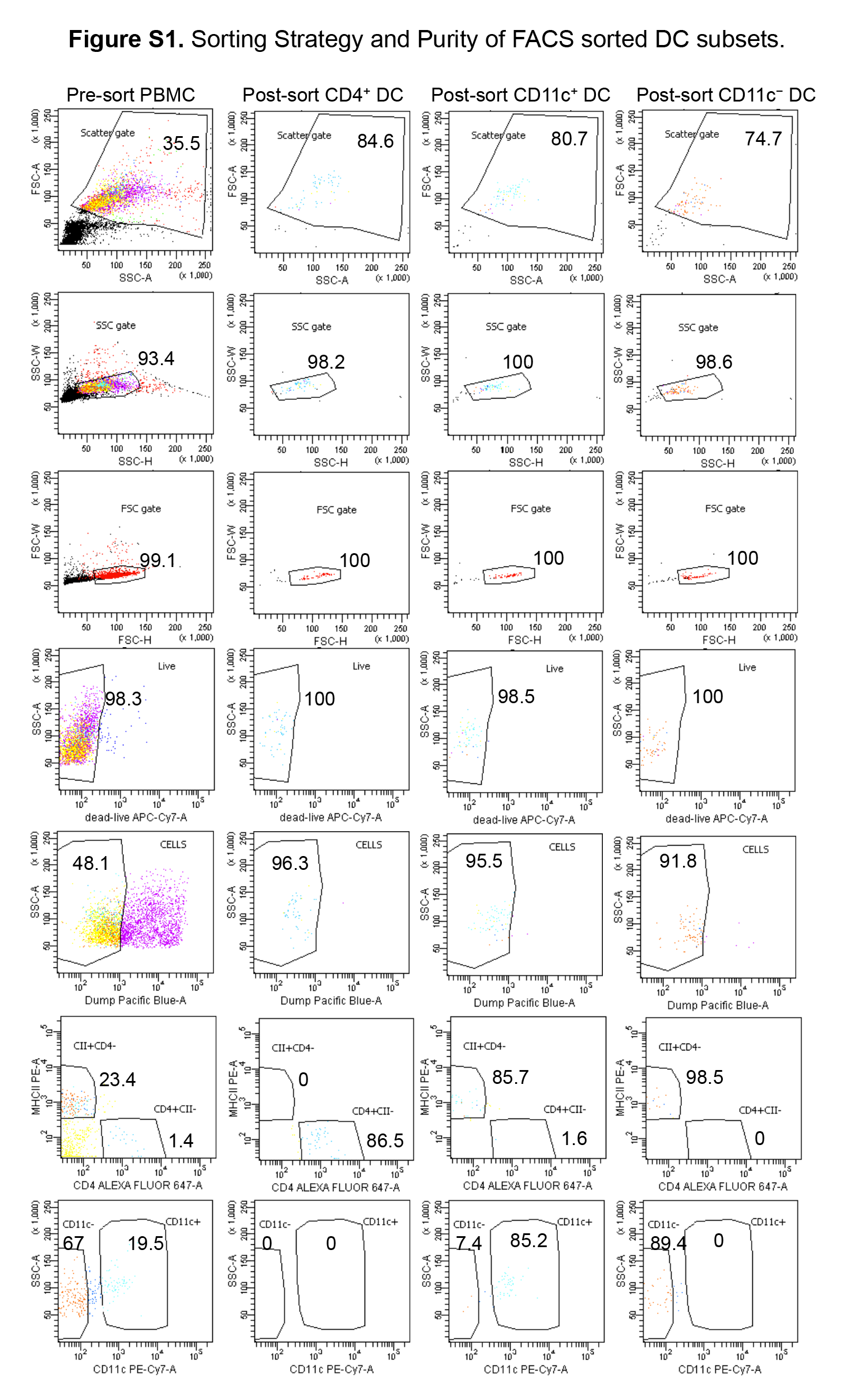

Supplement: Figure S1 — Sorting Strategy for DC subsets. PBMC were sorted on a FACS-Aria (BD, San Jose, CA) by gating on cells with appropriate forward and side scatter (rows 1 and 2), excluding dead cells (row 3), excluding lineage cells (row 4), and separating cells by expression of MHC class II, CD4, and CD11c (rows 5 and 6). Three populations were isolated as labeled; CD4+ DC, CD11c+ DC and CD11c- DC. The percent of the population isolated with the indicated phenotype are labeled in the dot plots. (TIF) [file pone.0109273.s001.tif]
